# A case of a renal abscess caused by *Salmonella* bareilly in a previously healthy boy

**DOI:** 10.1186/s12879-022-07229-1

**Published:** 2022-03-10

**Authors:** Tomomi Nakamura, Masaru Ido, Masahiro Ogawa, Naoya Sasaki, Haruna Nakamura, Yoshihiro Hasegawa, Motoki Bonno, Shigeki Tanaka

**Affiliations:** 1 Department of Paediatrics, National Hospital Organization Mie Chuo Medical Centre, 2158-5, Myojincho Hisai, Tsu, Mie Japan; 2 Clinical Research Institute, National Hospital Organization Mie Chuo Medical Centre, 2158-5, Myojincho Hisai, Tsu, Mie Japan; 3 Department of Paediatrics, National Hospital Organization Mie National Hospital, 357 Osatokubotacho, Tsu, Mie Japan; 4 Department of Urology, National Hospital Organization Mie Chuo Medical Centre, 2158-5, Myojincho Hisai, Tsu, Mie Japan

**Keywords:** *Salmonella* bareilly, Renal abscess, Broad-range 16S rDNA PCR, Single-nucleotide polymorphism

## Abstract

**Background:**

Renal abscesses are relatively uncommon in children, and usually due to Gram-negative rods or *Staphylococcus aureus*, whereas abscesses caused by *Salmonella* are very rare.

**Case presentation:**

We present the case of a previously healthy 10-year-old boy who had a renal abscess due to *Salmonella* bareilly. He responded well to treatment with antibiotics, and computed tomography (CT)-guided drainage of the abscess. His blood, urine and abscess aspirate cultures were sterile, but a broad-range 16S rDNA polymerase chain reaction (PCR) assay of the aspirate followed by analysis of four *Salmonella* genes (*fliC*, *fliD*, *sopE2*, and *spaO*) identified *S*. bareilly as the causative agent.

**Conclusion:**

To the best of our knowledge, this is the first report of renal abscess caused by *S.* bareilly.

## Background

Nontyphoidal salmonellae are foodborne and waterborne [1] pathogens that cause gastroenteritis, bacteraemia, and focal infection. Endovascular infection and deep bone or visceral abscesses are severe complications that may be difficult to treat [2]. However, renal abscess is a rare consequence of *Salmonella* bacteraemia, and the presence of a urogenital abnormality or compromised host immunity may predispose patients to complications even in cases of transient bacteraemia. *S.* bareilly, a group C1 serovar first identified in India in 1928 [3] is one of the most common *Salmonella* found in water [1] that can cause food poisoning. There was previously a worldwide foodborne outbreak of *S.* bareilly [4]. *S.* bareilly is less invasive than other nontyphoidal *salmonella* serovars [5] and has not, to our knowledge, been associated with renal abscesses.

Microbiological culture is an essential procedure for diagnosis of bacteraemia and sepsis, but prior use of antibiotics before sampling frequently reduces the detection rate of bacteria in culture studies. In these cases, molecular methods may be useful to identify the causative agent [6]. Here, we describe a case of renal abscess in a 10-year-old previously healthy boy who had no urogenital abnormality. The patient was previously treated with antibiotics, and thus, no bacterial growth was observed in the drainage culture. The application of a broad-range bacterial polymerase chain reaction (PCR) of 16S rDNA technology coupled with sequencing of four *Salmonella* genes identified *S.* bareilly as the causative agent of the infection.

## Case presentation

**Fig. 1:**
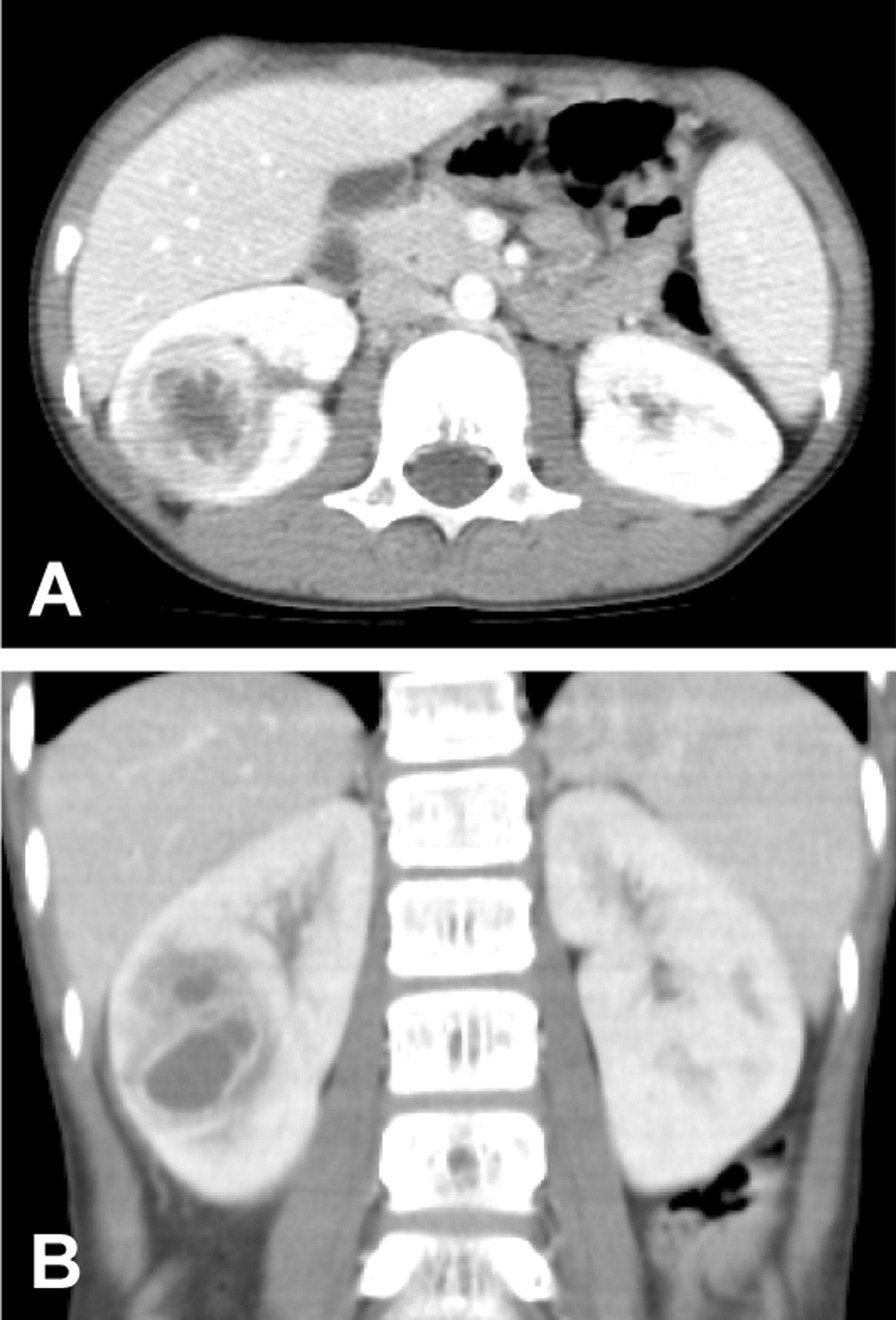
Abdominal contrast-enhanced computed tomography of the patient, **A** horizontal and **B** vertical slices

A 10-year-old previously healthy boy living in Japan presented with a 6-week history of fever, appetite loss, and weight loss of 2 kg. He had met normal developmental milestones and had received routine childhood vaccinations. He had no history of urinary tract infection or allergies. The family history was unremarkable. These symptoms began approximately 2 weeks after his return from Guam, where he had travelled for a 6-day vacation with his parents and brother. He enjoyed swimming at a pool in a “3-star resort hotel”, and frequently dived into the water. He received no typhoid vaccination or prophylactic therapy prior to his travel. He had no abdominal pain or diarrhoea but had precordial discomfort, difficulty swallowing, some nausea, and anorexia. Two weeks before admission, examination by a home doctor or otolaryngologist was unremarkable, except for tonsillar hypertrophy. One week before admission, he was found to have low-grade fever. Four days before admission, he was treated by a home doctor with azithromycin (10 mg/kg/day) for 3 days, but his symptoms did not improve. Physical examination on admission at the paediatric centre was unremarkable except for fever (38.5 °C) and right-sided flank tenderness. Pertinent laboratory findings included a total white blood cell (WBC) count of 8.35 (normal value, 3.5–8.5) × 10^9^/L, with 64% neutrophils. C-reactive protein (CRP) was 88.4 (normal value, 0.0–3.0) mg/L. Renal function tests revealed normal blood urea nitrogen (10.1 mg/dL) and serum creatinine (0.37 mg/dL). Urinalysis was normal. No organism was isolated from urine or blood culture. The test for human immunodeficiency virus was negative. Ultrasound examination showed that the right kidney was normal in size (9.8 × 4.2 cm) but had a cystic lesion (4.58 × 3.63 × 3.36 cm). The left kidney showed no evidence of disease. There was no abnormality in the urinary tract. Computed tomography (CT) revealed a cystic mass with ring enhancement in the right kidney (Fig. 1). Based on these results, we tentatively diagnosed his illness as a right renal abscess and treated him with ceftriaxone 2 g (80 mg/kg/day) by the intravenous route in two divided doses for 3 days. Because there was no clinical improvement, the treatment was changed to meropenem trihydrate (120 mg/kg/day), which was administered three times a day for 3 days. Thereafter, vancomycin (45 mg/kg/day) was added three times a day for 3 days because of increased fever (38.9 °C) and CRP (114.8 mg/L). However, because there was still no clinical improvement despite a 10-day treatment with antibiotics, he was transferred to our hospital, where under local anaesthesia, a CT-guided percutaneous puncture of the renal abscess was performed, and 10 mL of creamy pus was drained. He became afebrile soon after, and the flank tenderness also subsided, but he was continued on the antibiotic regimen of intravenous meropenem and vancomycin for an additional 15 days. The duration of hospitalization was a total of 31 days. He was thereafter discharged, and remained asymptomatic for more than 1 year.

**Fig. 2:**
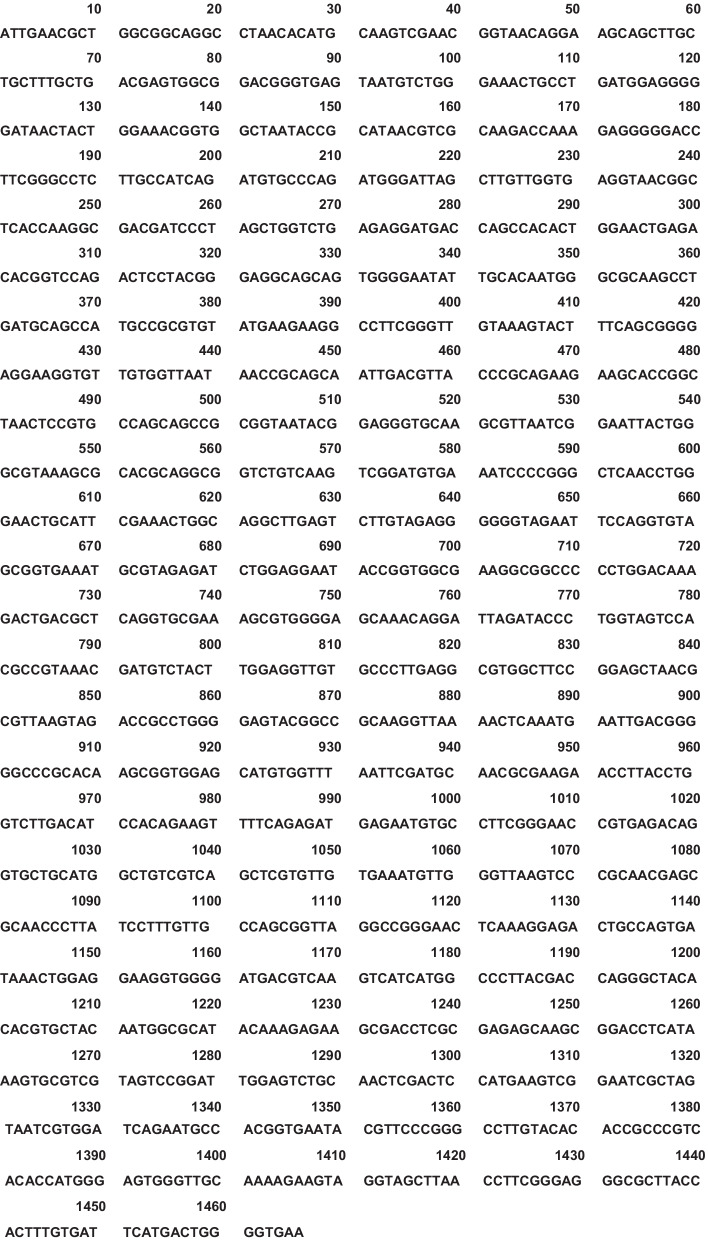
DNA sequence of the 16S rDNA PCR product amplified using DNA extracted from an aspirate as a template and 8UA and 1485B primers

Gram staining of the aspirate revealed only WBCs, and its culture was sterile. Therefore, broad-range 16S rDNA PCR amplification and sequencing were performed using PCR primers 8UA (5′-AGAGTTTGATCMTGGCTCAG-3′) and 1485B (5′-TACGGTTACCTTGTTACGAC-3′) [7] and DNA extracted from the aspirate. The sequence (Fig. 2) was 99.9% identical (1465/1466) to several *Salmonella enterica* strains.

**Fig. 3:**
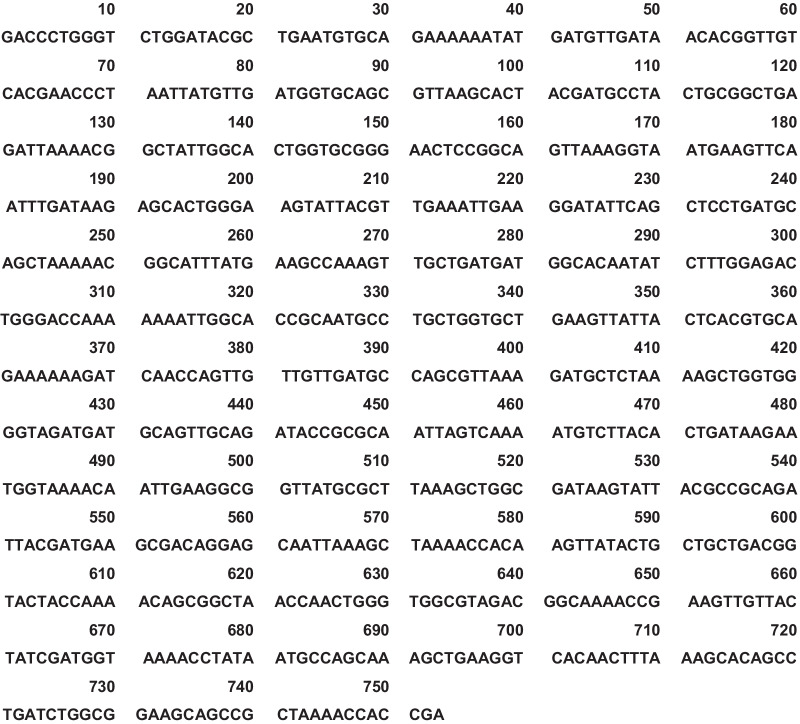
DNA sequence of the *fliC* PCR product amplified using DNA extracted from an aspirate as a template and *fliC* forward and reverse primers

To identify the serovar of *Salmonella*, we examined single-nucleotide polymorphisms (SNPs) located on three *Salmonella* genes, *fliD* (STM1960), *sopE2* (STM1855), and *spaO* (STM2891) [8], and found SNPs identical to *S.* bareilly or *S.* paratyphi B variant Java (Table 1). Next, we examined the *fliC* gene of *S.* bareilly and *S.* paratyphi B variant Java by comparison with that of *S*. typhimurium (STM1959). Further PCR amplification and sequencing were also performed to analyse the *fliC* gene using forward (5′-CGATCTGAAGCAGATCAACTCTCA-3′) and reverse (5′-CATCAATTTTAGCCAGCGGGTTT-3′) primers and DNA extracted from the aspirate. The sequence of the PCR product (Fig. 3) was 100% (753/753) identical to that of *S.* bareilly strain CFSAN000189 and 73.9% (557/753) identical to *S.* paratyphi B variant Java strain 08-00436.

**Table 1:** Twenty-three SNPs analysed by sequencing three genes

	*fliD*										*sopE2*							*spaO*					
*Salmonella enterica* serovar	612*	616	639	651	747	748	753	758	765	786	421	455	463	465	471	477	480	68	73	80	87	156	163
*S.* bareilly	C	C	C	T	A	A	G	C	C	C	C	G	G	G	A	T	T	A	C	G	G	G	C
*S.* paratyphi B variant Java	C	C	C	T	A	A	G	C	C	C	C	G	G	G	A	T	T	A	C	G	G	G	C
Patient	C	C	C	T	A	A	G	C	C	C	C	G	G	G	A	T	T	A	C	G	G	G	C

The results of *S.* bareilly and *S.* paratyphi B variant Java are as reported [8]

*The numbers represent the nucleotide location on the coding gene

We performed follow-up cultures of blood, urine, and aspirate but did not detect any bacteria. We also attempted to detect *S*. bareilly by broad-range 16S rDNA PCR using blood specimens drawn 4 days and 1 month after admission, but the results were negative.

## Discussion and conclusion

Renal abscesses, potentially lethal complications of urinary tract infections or bacteraemia, are infrequently encountered in children [9–11], but the actual prevalence is still unknown [10]. Paediatric renal abscesses are most commonly small in size and less than 3 cm in diameter [10, 11]. The most common pathogens isolated in children are *Escherichia coli* and *Staphylococcus aureus* [9–11]. According to a previous report, broad-spectrum and bactericidal antibiotics, such as second- and third-generation cephalosporins, are recommended for children with renal abscesses [12]. However, pathogens are sometimes resistant to antibiotic treatment [12, 13]. Thus, for initial treatment, antibiotics, such as meropenem plus vancomycin, should be selected, considering the harmful and destructive effects of renal abscesses on the kidneys. This combination is effective against not only Gram-negative bacilli but also Gram-positive bacteria, even though they are resistant strains.

There are no definite guidelines for surgical intervention in paediatric patients [14]. In adult patients, abscesses of less than 3 cm in diameter usually resolve with antibiotic therapy for 4–6 weeks [15]. Furthermore, several recent studies on paediatric renal abscesses have reported successful effects of antibiotic therapy against abscesses of less than 3 cm in diameter [10, 11]. Percutaneous drainage may be considered for lesions of more than 3 cm in patients with persistent fever despite treatment with appropriate antibiotics or in patients who are immunologically compromised or critically ill [9–11]. In our case, the lesion was more than 3 cm, and the abscess was refractory to antibiotic therapy, and therefore required drainage. The prompt resolution of our patient’s clinical symptoms after drainage suggests that surgical intervention is helpful.

*Salmonella* could cause gastroenteritis, bacteraemia, and subsequent focal infections. However, invasive nontyphoidal salmonellosis rarely affects the kidneys, and there are only a small number of reports of renal abscesses caused by *Salmonella* serovars, such as *S.* virchow [16], *S.* enteritidis, *S.* typhimurium [17, 18], and *S*. oranienburg [19]. Also, some nontyphoidal *Salmonella* serovars, such as *S.* choleraesuis and *S.* dublin, can cause more invasive disease than *S.* typhimurium or *S.* bareilly [5]. Indeed, there are only a small number of reports of extraintestinal infections by *S.* bareilly [20, 21].

The route of *Salmonella* infection in our patient is unclear, although infectious agents have been reported to threaten the health of pool users in tropical countries [22–24]. Since his family members did not show gastrointestinal symptoms during and after their trip, we speculate that he may have ingested contaminated water from the pool in the hotel. Unfortunately, we could not obtain information on *Salmonella* outbreaks associated with pool water in Guam.

We were unable to isolate the microorganisms from the abscess, perhaps because he had been on antibiotics sometimes before the sample was taken for culture. The diagnosis was therefore based on the result of broad-range PCR [6, 7], which can detect a wide variety of bacteria from biological samples even after sterilization with antibiotics. However, this alone is insufficient to determine the *Salmonella* serovar, and additional examination for specific bacterial genes [8] was required.

We conclude, to the best of our knowledge, that this is the first report of a paediatric renal abscess due to *S.* bareilly, which was detected using broad-range PCR followed by DNA sequencing of specific bacterial genes. The child responded satisfactorily to treatment with antibiotics and percutaneous surgical drainage of the abscess.

## Data Availability

The datasets generated and analysed during the current study are available in the DNA Data Bank of Japan (DDBJ) repository under the following Accession Numbers: LC687364, LC687365.

## Abbreviations

CT: Computed tomography
DNA: Deoxyribonucleic acid
PCR: Polymerase chain reaction
rDNA: Ribosomal DNA
WBC: White blood cell
CRP: C-reactive protein
SNP: Single-nucleotide polymorphism
